# CSF-S100B Is a Potential Candidate Biomarker for Neuromyelitis Optica Spectrum Disorders

**DOI:** 10.1155/2018/5381239

**Published:** 2018-10-22

**Authors:** Yuzhen Wei, Haoxiao Chang, Xindi Li, Li Du, Wangshu Xu, Hengri Cong, Yajun Yao, Xinghu Zhang, Linlin Yin

**Affiliations:** ^1^Department of Neurology, Beijing Tiantan Hospital, Capital Medical University, Beijing 100050, China; ^2^China National Clinical Research Center for Neurological Diseases, Beijing 100050, China

## Abstract

Astrocytic impairment is a pathologic feature of neuromyelitis optica spectrum disorder (NMOSD). S100B and glial fibrillary acidic protein (GFAP) are the two most commonly used astrocytic markers. The aim of this study was to evaluate whether CSF-S100B could serve as a marker of NMOSD. We enrolled 49 NMOSD patients [25 aquaporin-4 antibody (AQP4-Ab)–positive, 8 myelin-oligodendrocyte glycoprotein antibody (MOG-Ab)-positive, and 16 seronegative patients], 12 multiple sclerosis (MS) patients, and 15 other noninflammatory neurological diseases (OND) patients. The CSF levels of S100B and GFAP were measured by ELISA. Both CSF-S100B and GFAP levels significantly discriminated NMOSD from MS [area under curve (AUC) = 0.839 and 0.850, respectively] and OND (AUC = 0.839 and 0.850, respectively). The CSF-S100B levels differentiated AQP4-Ab–positive NMOSD from MOG-Ab–positive NMOSD with higher accuracy than the CSF-GFAP levels (AUC=0.865 and 0.772, respectively). The CSF-S100B levels also significantly discriminated MOG-Ab–positive patients from seronegative patients (AUC = 0.848). Both CSF-S100B and GFAP levels were correlated with the Expanded Disability Status Scale (EDSS) during remission. Only the CSF-S100B levels were correlated with the CSF WBC count and the EDSS during attack. The levels of CSF-S100B seemed to have a longer lasting time than the levels of CSF-GFAP, which may benefit patients who present late. As a result, CSF-S100B might be a potential candidate biomarker for NMOSD in discriminating, evaluating severity, and predicting disability.

## 1. Introduction

Neuromyelitis optica spectrum disorder (NMOSD) is a relapsing and often severely disabling autoimmune disease of the central nervous system (CNS), predominantly targeting the optic nerves and spinal cord [1]. More than half of the patients with NMOSD are positive for autoantibodies against the water channel aquaporin-4 (AQP4-Ab), which is mainly expressed in astrocytic foot processes [2, 3]. Astrocytic impairment associated with the loss of AQP4 is a pathologic feature of NMOSD, which is distinct from multiple sclerosis (MS) [4].

S100B and glial fibrillary acidic protein (GFAP) are two astrocytic markers often used to indicate astrocytic damage or dysfunction [5]. In the cerebrospinal fluid (CSF) of patients with neuromyelitis optica (NMO), the levels of S100B and GFAP are higher than those in the CSF of patients with MS and other noninflammatory neurological disorders (OND) and correlate with Expanded Disability Status Scale (EDSS) during attack and the length of spinal cord lesion [6, 7]. However, CSF-S100B is considered to be less astrocyte-specific than GFAP [8]. To clarify whether CSF-S100B could serve as a potential marker for NMOSD patients, in the present study, we compared the discriminating value of CSF-GFAP and S100B levels for NMOSD and its subtypes. In addition, the correlations of these markers with clinical and laboratory data have also been evaluated.

## 2. Methods

### 2.1. Patients

Patients with NMOSD and MS were recruited from the Beijing Tiantan Hospital between March 2016 and September 2017. The NMOSD and MS diagnoses were made according to 2015 Revised International Criteria [9] and 2010 McDonald's Diagnostic Criteria [10], respectively. Patients who met the following three conditions were included: (1) the CSF samples were collected during the acute phase (within 30 days of the symptom onset; or for patients who experienced exacerbations within 3 weeks of onset, the CSF were collected within 30 days of the exacerbations) and before any immunotherapy; (2) there were no infectious or other autoimmune comorbidities at the time of sample collection; (3) clinical characteristics, including gender, age, routine CSF [white blood cell (WBC) count, protein level, IgG index] and MRI information, and the EDSS disability score during attack and remission were prospectively recorded. In addition, 15 patients with OND were enrolled (13 women and 2 men; mean age 40.2 years). The OND group included patients with benign intracranial hypertension (n=3), cluster headache (n=3), psychogenic movement disorders (n=3), normal pressure hydrocephalus (n=2), benign paroxysmal positional vertigo (n=2), sleep disturbance (n=1), and vitamin B12 deficiency (n=1).

The study was approved by the Ethics Committee of Beijing Tiantan Hospital affiliated with Capital Medical University, Beijing, China (No. KY2015-031-02), and written informed consent was obtained from all participants.

### 2.2. Biomarker Measurement

The CSF samples were centrifuged, and the supernatants were collected and stored at −80°C until analysis. Positivity for AQP4-Ab and MOG-Ab was determined using the cell-based assay (CBA) with live HEK-293 cells transiently transfected with full-length M23-AQP4 or the plasmid containing full-length human MOG, as described previously [11, 12]. The levels of CSF-S100B and GFAP were measured by ELISA: S100B (EZHS100B-33K, Milliplex Merck KGaA, Darmstadt, Germany), GFAP (NS830, Milliplex Merck KGaA, Darmstadt, Germany). The detection limit was 2.7 pg/ml for S100B and 1.5 ng/ml for GFAP. All samples were assayed in duplicate, and all testing was performed according to the manufacturer's protocols and in a manner blinded to the diagnosis or clinical presentations.

### 2.3. Statistical Analysis

Statistical analysis was conducted using SPSS 22.0 (International Business Machines Corporation, Chicago, IL, USA). For comparison among groups, the categorical data were compared with Fisher's exact test. Continuous data were compared with the nonparametric Mann–Whitney U test with Bonferroni correction. A two-tailed Spearman's rank correlation coefficient was used to ascertain the associations. We judged correlations as strong when the correlation coefficients (r) were > 0.6. Receiver operating characteristic (ROC) curves were used to evaluate the discriminating values. The Youden index was calculated to determine the cutoff value. A two-tailed* p* value <0.05 was considered statistically significant.

## 3. Results

### 3.1. Clinical Demographics

Forty-nine patients with NMOSD and 12 patients with MS were included. Among the NMOSD patients, 51.0% (25/49) were positive for AQP4-Ab, 16.3% (8/49) were positive for MOG-Ab, and the remaining 32.7% (16/49) were seronegative for both MOG-Ab and AQP4-Ab. No patients were positive for both antibodies. The demographic and clinical features of the patients are summarized in Table 1. The EDSS score of all NMOSD patients was 6 (2-8.5) [median (range)], which was significantly higher than that of the MS patients (*p* < 0.001). Among the NMOSD patients, there was a significantly higher proportion of males in the MOG-Ab–positive group than the AQP4-Ab–positive group (*p* = 0.01), which was consistent with previous studies [13–15]. There were no significant differences in the sex ratio among the other groups. No significant differences were observed regarding the age or disease duration between NMOSD with MS or among subgroups of NMOSD.

### 3.2. Discriminating Value of CSF-GFAP and CSF-S100B Levels for NMOSD, MS, and OND

**Figure 1:**
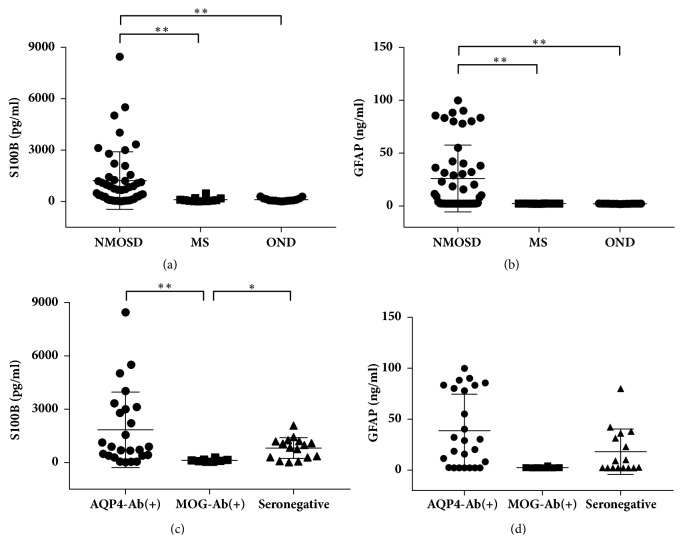
CSF levels of S100B and glial fibrillary acidic protein (GFAP) in neuromyelitis optica spectrum disorder (NMOSD), multiple sclerosis (MS), other noninflammatory neurological diseases (OND), and NMOSD subgroups. (a) Patients with NMOSD had significantly higher levels of CSF-S100B than those with MS and OND. (b) NMOSD patients had significantly higher levels of CSF-GFAP than MS and OND patients. (c) The CSF-S100B levels in aquaporin-4 antibody-positive NMOSD [AQP4-Ab (+)] and seronegative NMOSD were significantly higher than those in myelin-oligodendrocyte glycoprotein antibody-positive NMOSD [MOG-Ab (+)]. (d) The CSF-GFAP levels were higher in AQP4-Ab (+) patients than those in MOG-Ab (+) patients but did not reach a significant difference. Each dot represents a biomarker level in a subject. Lines and whiskers represent mean values and standard deviation, respectively. *∗p* < 0.05 and *∗∗p* < 0.01 represent statistical significance in the Mann–Whitney U test with Bonferroni correction.

The levels of S100B and GFAP in the CSF of NMOSD patients during the acute phase were higher than levels in the CSF of MS (both* p*<0.001) and OND patients (both* p*<0.001). No significant differences were found in the levels of S100B and GFAP between the MS and OND groups (*p*=0.683 and* p*=0.139, respectively) (Figures 1(a) and 1(b)). To assess the discriminating value of CSF-GFAP and CSF-S100B levels for NMOSD with MS and OND, we performed a ROC analysis (Table 2). The S100B and GFAP levels significantly discriminated NMOSD from MS [area under curve (AUC) = 0.839 and 0.850, respectively]. The optimal cutoff points for S100B and GFAP levels were 241.4 pg/ml and 2.3 ng/ml, respectively (Supplementary Figure  1a). The sensitivity (71.4%) and specificity (91.7%) were the same when using the optimal cutoff points for S100B and GFAP to discriminate NMOSD from MS. The S100B and GFAP levels could also significantly discriminate NMOSD from OND (AUC = 0.839 and 0.850, respectively) but failed to discriminate MS from OND (AUC = 0.450 and 0.669, respectively) (Supplementary Figures  1b, c).

### 3.3. Discriminating Value of CSF-GFAP and CSF-S100B Levels for AQP4-Ab–Positive, MOG-Ab–Positive, and Seronegative NMOSD Patients

In NMOSD patients, the levels of CSF-S100B were significantly higher in AQP4-Ab–positive and seronegative patients than in MOG-Ab–positive patients (*p* < 0.01 and* p *< 0.05, respectively) (Figure 1(c)). The CSF-GFAP levels in AQP4-Ab–positive patients were higher than those in MOG-Ab–positive patients but did not reach a significant difference (*p* = 0.020 < 0.05/3). No significant differences were found in the CSF-GFAP levels between the seronegative and MOG-Ab–positive patients (Figure 1(d)).

We next determined the discriminating value of CSF-S100B and CSF-GFAP levels for different subtypes of NMOSD (Table 2) (Supplementary Figure  1). Both CSF-S100B and CSF-GFAP levels significantly differentiated patients with AQP4-Ab–positive NMOSD from those with MOG-Ab–positive NMOSD (AUC = 0.865 and 0.772, respectively). The CSF-S100B levels also significantly discriminated MOG-Ab–positive patients from seronegative patients (AUC = 0.848).

### 3.4. Correlations of CSF-GFAP and S100B Levels with Clinical and Laboratory Data in NMOSD Patients

**Figure 2:**
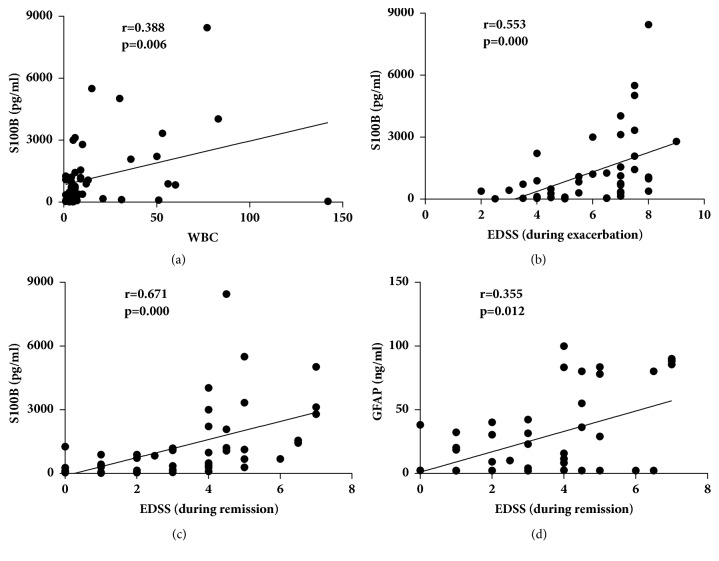
Positive correlations between the CSF-S100B or glial fibrillary acidic protein (GFAP) levels and clinical/laboratory findings in neuromyelitis optica spectrum disorder (NMOSD) patients. The CSF-S100B levels correlated with the CSF white blood cell (WBC) count (a), EDSS during attack (b), and during remission (c). The CSF-GFAP levels correlated with the EDSS during remission (d). Statistical testing was performed by using Spearman's rank correlation analysis. r = Spearman's rho.

In NMOSD patients, the CSF-S100B levels correlated with the CSF WBC count (r = 0.388,* p* = 0.006) and the EDSS during attack and during remission (r = 0.553,* p* < 0.001, and r = 0.671,* p* < 0.001, respectively) (Figure 2). The CSF-GFAP levels correlated with the EDSS during remission (r = 0.355,* p* = 0.012). No significant correlations of the CSF-S100B and GFAP levels with the changes in EDSS, CSF protein levels, IgG index, number of T2 lesions and gadolinium enhancing lesions in the brain and spinal cord were found in NMOSD patients.

In AQP4-Ab–positive NMOSD patients, the AQP4 titer did not correlate with the CSF-S100B or GFAP levels (r = -0.061,* p* = 0.772, and r = -0.156,* p* = 0.456, respectively).

Attack-related lesions were found in the spinal cord without concomitant cerebrum or brainstem lesions in six patients (2 AQP4-Ab–positive NMOSD, 1 MOG-Ab–positive NMOSD and 3 seronegative NMOSD). Because of the small sample size and different subgroups, we did not find correlations between the lengths of longitudinal spinal lesions and the CSF-S100B or GFAP levels (r =0.145,* p* = 0.784, and r = 0.145,* p* = 0.784, respectively) in these patients.

### 3.5. Correlations between the CSF-GFAP and S100B Levels in NMOSD, MS, and NMOSD Subgroups

**Figure 3:**
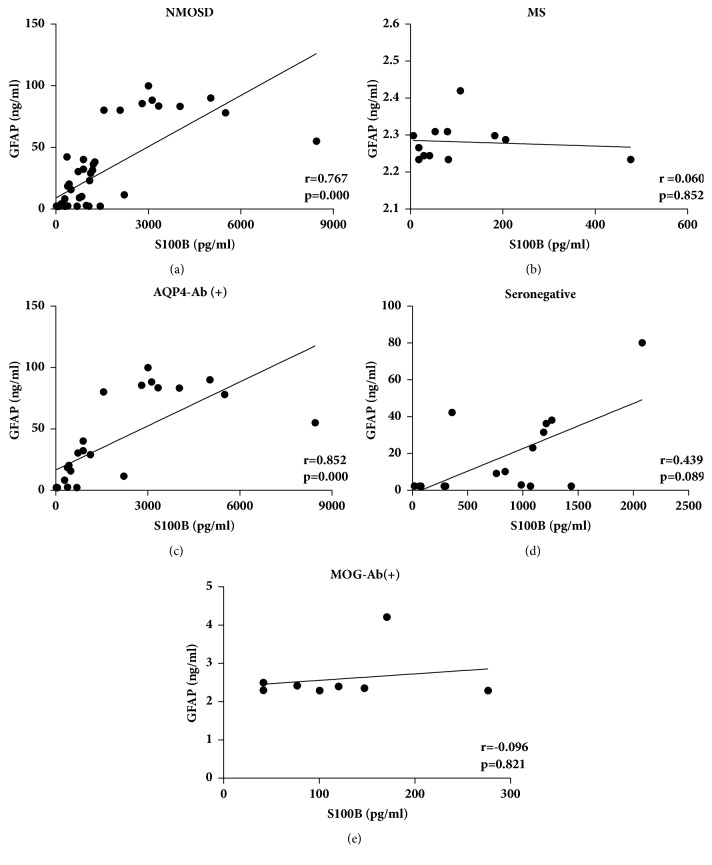
Correlations between CSF-GFAP and S100B levels in NMOSD, MS, and NMOSD subgroups. In NMOSD patients, the CSF-S100B and GFAP levels were strongly correlated with each other (a). In MS patients, the CSF-S100B levels did not correlate with the GFAP levels (b). Among NMOSD subgroups, the CSF-S100B and GFAP levels strongly correlated with each other in AQP4-Ab-positive NMOSD [AQP4-Ab (+)] patients (c); in seronegative (d) and MOG-Ab-positive NMOSD [MOG-Ab (+)] patients (e), the CSF-S100B levels did not correlate with the GFAP levels. Statistical testing was performed by using Spearman's rank correlation analysis. r = Spearman's rho.

First, we analyzed the correlations between CSF-GFAP and S100B levels in NMOSD and MS patients. In NMOSD patients, the CSF-S100B and GFAP levels strongly correlated with each other (r = 0.767,* p* < 0.001) (Figure 3(a)). In MS patients, the CSF-S100B levels did not correlate with the GFAP levels (r =0.060,* p* = 0.852) (Figure 3(b)). Among NMOSD subgroups, the CSF-S100B and GFAP levels were strongly correlated with each other in AQP4-Ab-positive NMOSD patients (r = 0.852,* p* < 0.001) (Figure 3(c)). However, in seronegative and MOG-Ab-positive NMOSD patients, the CSF-S100B levels did not correlate with the GFAP levels (r =0.439,* p* = 0.089, and r = -0.096,* p* = 0.821, respectively) (Figures 3(d) and 3(e)).

**Figure 4:**
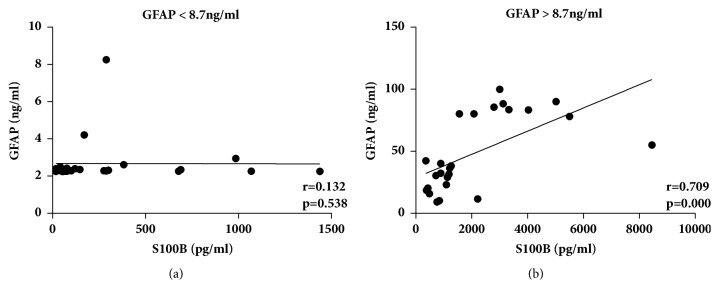
Correlation between CSF-S100B and glial fibrillary acidic protein (GFAP) levels in neuromyelitis optica spectrum disorder (NMOSD) patients. In the GFAP < 8.7 ng/ml group, the CSF-S100B levels did not correlate with the GFAP levels (a); in the GFAP > 8.7 ng/ml group, the CSF-S100B and GFAP levels strongly correlated with each other (b). Statistical testing was performed by using Spearman's rank correlation analysis. r = Spearman's rho.

Second, we analyzed the correlations between CSF-GFAP and S100B levels in NMOSD patients with different levels of CSF-GFAP. We divided the NMOSD patients into two groups according to the median CSF-GFAP level (8.7 ng/ml). In the GFAP < 8.7 ng/ml group, the CSF-S100B levels did not correlate with the GFAP levels (r = 0.132,* p* = 0.538) (Figure 4(a)). However, in the GFAP > 8.7 ng/ml group, the CSF-S100B and GFAP levels were strongly correlated with each other (r = 0.709,* p* < 0.001) (Figure 4(b)).

### 3.6. Influence of Intervals between Attack Onsets and Lumbar Punctures on CSF-GFAP and CSF-S100B Levels in NMOSD Patients

**Figure 5:**
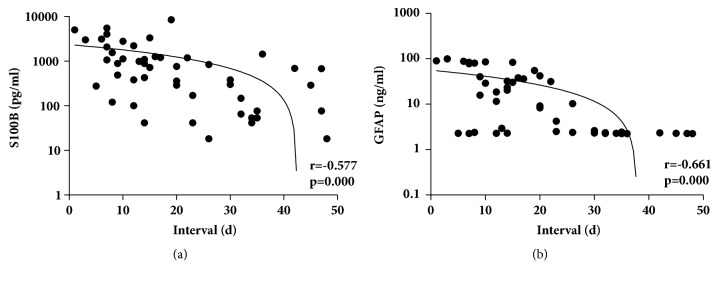
Relation of CSF-S100B and CSF-glial fibrillary acidic protein (GFAP) levels with the intervals between relapse onset and lumbar puncture in neuromyelitis optica spectrum disorder (NMOSD) patients. The CSF-S100B (a) or CSF-GFAP (b) levels were both significantly related to the intervals between attack onsets and lumbar punctures. Statistical testing was performed by using Spearman's rank correlation analysis. r = Spearman's rho.

The intervals between attacks and lumbar punctures (days, mean±SD) were 18.4±14.0 in AQP4-Ab–positive NMOSD, 23.6±12.0 in seronegative NMOSD, 19.0±11.0 in MOG-Ab–positive NMOSD, and 24.3±8.4 in MS. There was no significant difference among the patient groups. We further speculated the changing patterns of CSF-S100B and CSF-GFAP through analyzing the correlations of their levels and the intervals from symptom onset to CSF sample collection in different NMOSD patients (Figure 5). The CSF-S100B or CSF-GFAP levels were both significantly related to the intervals between attack onsets and lumbar punctures (r = -0.577,* p* < 0.001 and r = -0.661,* p* < 0.001, respectively). The CSF obtained earlier after attack onsets tended to have higher levels of both S100B and GFAP. However, the levels of CSF-S100B seemed to decrease more slowly than those of CSF-GFAP. At 27 days after attack onsets, the levels of CSF-GFAP in most patients became undetectable. However, many patients had relatively high levels of CSF-S100B, even at 40 days after symptom onset.

### 3.7. Influence of the Attack-Related Lesion Site on CSF-S100B and CSF-GFAP Levels in NMOSD Patients

In six patients with only spinal cord lesions and without concomitant cerebrum or brainstem lesions, the levels of CSF-S100B and GFAP [median (range)] were 684.1 (276.7-3124.3) pg/ml and 2.8 (2.3-88.3) ng/ml, respectively. Four patients had attack-related lesions in the cerebrum or brainstem without concomitant spinal cord lesions (2 MOG-Ab–positive NMOSD and 2 seronegative NMOSD). The levels of CSF-S100B and GFAP [median (range)] in these patients were 76.9 (65.1-100.3) pg/ml and 2.3 (2.3-2.4) ng/ml, respectively. The CSF-S100B and GFAP levels were higher in patients who had only spinal cord lesions than in those who had only cerebrum or brainstem lesions (*p* =0.010 and* p* =0.038, respectively). No patients had only optic nerve lesions in this series.

## 4. Discussion

GFAP is an intermediate filament protein, which is one of the key elements of the cytoskeleton of astrocytes [16, 17]. S100B, belonging to a family of calcium-binding proteins, is predominantly expressed in astrocytes [18, 19]. Both GFAP and S100B are common astrocytic markers. However, as an astrocytic damage biomarker, CSF-S100B is considered to be less specific than GFAP for the following reasons: (1) S100B is not only an astrocytic damage biomarker but also a glial modulator which constantly secreted from astrocytes, and implicated in intracellular and extracellular regulatory activities [18, 20]; (2) S100B is primarily an astrocytic protein, but it is also localized in many other neural cell types, such as oligodendrocytes, ependymal cells, and so on [8, 19]; and (3) the elevation of S100B levels is also found in malignant melanoma and other central nervous system tumors [21]. Therefore, CSF-S100B has many putative cellular sources. These reasons limit the application of S100B in clinical practice. In this small-scale study, we provided a new sight for clinical values of CSF-S100B, showing its advantages in differentiating subgroups of NMOSD patients and clinical relevance.

Recent studies have shown that the CSF levels of GFAP were higher in NMO patients than in MS and OND patients. CSF-S100B levels showed a trend similar to that of GFAP levels but were less remarkable [6, 7]. In line with previous studies, we also found that NMOSD patients exhibited significantly elevated levels of CSF-GFAP and S100B than MS and OND patients. In addition, we provide evidence that the CSF levels of both GFAP and S100B might represent useful markers for discriminating NMOSD patients from MS and OND patients with high accuracy. The discriminating accuracy of CSF-GFAP was slightly higher than that of CSF-S100B, but at the optimum cutoff points, these markers had the same or similar sensitivity and specificity. As a result, CSF-S100B could be a candidate marker for distinguishing NMOSD from MS and OND.

A total of 78-83% of NMOSD patients have AQP4-Ab [22]. Approximately 42% of AQP4-Ab-negative NMOSD patients are positive for MOG-Ab [23]. In addition to NMOSD, MOG-Ab is also identified in other demyelinating diseases, including some cases of acute disseminated encephalomyelitis (ADEM), multiphasic demyelinating encephalomyelitis, MS, optic neuritis (ON), and longitudinally extensive transverse myelitis [15, 24–28]. MOG-Ab-associated diseases and AQP4-Ab-positive NMOSD share some clinical phenotypes, but MOG-Ab-associated diseases do have some distinct clinical features from AQP4-Ab-positive NMOSD: a higher proportion of males, fewer relapses, better recovery, more bilateral simultaneous ON, more symptomatic brain disease appears manifest as seizures or encephalitis, and a wider spectrum of MRI features [13, 29–34]. In addition, the injuries induced by MOG-Ab and AQP4-Ab are different. The astrocytic impairment and increased permeability of the blood–brain barrier (BBB) by AQP4-Ab result in the leakage of GFAP in AQP4-Ab-positive NMOSD [4, 6, 35]. In MOG-Ab-positive NMOSD, MOG-Ab binds MOG and damages oligodendrocytes or myelin, leading to the release of myelin basic protein (MBP), but astrocyte injury with GFAP elevation is absent [27, 36, 37]. In accordance with this finding, our study also found that CSF-S100B and GFAP levels were higher in AQP4-Ab-positive NMOSD patients than those in MOG-Ab-positive NMOSD patients. Therefore, the term MOG-Ab-positive NMOSD represents a fundamental disconnect from our current understanding of NMO as an astrocytopathy and should be set apart from AQP4-Ab-positive NMOSD [31]. It is important to identify the role of MOG-Ab-associated diseases in idiopathic inflammatory demyelination, either as a subtype of other demyelinating diseases or as a separate disease entity, not only for further understanding of the pathogenesis but also has practical implications for therapy [31].

Furthermore, in this study, we demonstrated for the first time that seronegative NMOSD patients had middle levels of S100B and GFAP (Figures 1(c) and 1(d)). Although it is unclear whether seronegative NMOSD is the same autoimmune astrocytopathic disease as AQP4-Ab-positive NMOSD [38], the elevation of CSF-S100B and GFAP levels indicated the presence of astrocytic damage. As seronegative NMOSD patients could be heterogeneous, a subgroup of these individuals might be associated with other autoantibodies like AQP4-Ab, which targeted astrocytes and caused the leakage of S100B and GFAP [39, 40]. Because different subtypes of NMOSD had different degrees of astrocytic injury, we hypothesized that the astrocytic markers might discriminate between disease subtypes of NMOSD. In our study, both the levels of CSF-S100B and GFAP could differentiate AQP4-Ab-positive NMOSD from MOG-Ab-positive NMOSD with high accuracy. CSF-S100B could also differentiate seronegative NMOSD from MOG-Ab-positive NMOSD. However, both biomarkers failed to distinguish AQP4-Ab-positive NMOSD from seronegative NMOSD. The CSF-S100B levels showed advantages in differentiating MOG-Ab-positive NMOSD from the other subtypes of NMOSD. Therefore, we suggest that although the CSF-S100B level cannot distinguish all subtypes of NMOSD independently, it can be used as a supplementary marker for auxiliary diagnosis.

In terms of clinical relevance, the CSF-S100B levels showed a significant correlation with the CSF WBC count and the EDSS during attack and during remission. The CSF-GFAP levels were only weakly correlated with the EDSS during remission. This finding suggested that CSF-S100B might be a better tool than CSF-GFAP for assessing the inflammatory activity and clinical severity of NMOSD, which was different from a previous study by Takano et al. [6]. The differing results between Takano's study and our study might be due to differences in patient characteristics. Takano's study included only AQP4-Ab-positive NMOSD patients, while we also included AQP4-Ab-negative patients. Another possible reason might be the different intervals between relapse onset and lumbar puncture. In both studies, patients whose CSF obtained earlier during relapse tended to have higher levels of GFAP. In our study, the CSF levels of S100B were also related to the intervals between attack onsets and lumbar punctures but did not decline as rapidly as GFAP. The intervals in Takano's study were shorter than those in our study. Approximately 27 days after attack onsets, the levels of CSF-GFAP in most patients became undetectable (Figure 5(b)). The strength of the correlations between CSF-GFAP and clinical data might be not as significant as before. The CSF-S100B levels decreased more slowly than the levels of CSF-GFAP probably because in addition to an astrocytic injury biomarker, S100B was also a modulator, which was one of the Danger/Damage Associated Molecular Pattern (DAMP) molecules. The secretion of S100B was increased during the glial response to brain injuries to trigger tissue reaction to damage [41–43]. S100B overproduction occurs in the early presymptomatic stage and during the whole course of the disease, which makes the elevation of CSF-S100B levels last longer than the elevation of CSF-GFAP levels. The elevation of CSF-S100B levels, both as an astrocytic biomarker or a modulator, can be used as a predictor of poor outcome. As a biomarker of astrocytic impairment, high levels of CSF-S100B indicate severe damage to astrocytes. As a modulator, S100B shows toxic/proinflammatory effects at high concentrations, leading to neuronal dysfunction or cell death because of an inflammatory response that stimulates astrocytes and microglia to recruit and produce proinflammatory cytokines with a subsequent increase of the extracellular levels of calcium and activation of nitric oxide [19, 44–47]. In both* ex vivo* demyelinating model and EAE-induced rats, the inhibition of S100B action using an anti-S100B neutralizing antibody has been shown to reduce demyelination, downregulate the expression of inflammatory molecules and improve the clinical course of the disease [48, 49]. A recent study showed that excessive S100B levels impaired oligodendrogenesis, resulting in reduced myelination [50]. The above data indicate that S100B plays a relevant role in the pathogenic mechanisms of demyelinating diseases. The elevation of the levels of CSF-S100B, both as an astrocytic biomarker or as a modulator, is of important value in monitoring the trend of the disorder and predicting clinical outcomes. Therefore, as CSF-GFAP declines rapidly to undetectable levels, the clinical correlations are weakened along with it, which may not be suitable for NMOSD patients who present late. In this case, the long-lasting CSF-S100B levels may be more appropriate than the GFAP levels for differentiating diseases, evaluating severity and predicting disability.

To our knowledge, limited studies have focused on the correlations between CSF-GFAP and S100B levels in NMOSD patients [6, 7], and few reports have analyzed their relation in subgroups or at different concentrations. In this study, we systemically investigated the correlations of CSF-S100B and GFAP levels in NMOSD subgroups and MS patients. We found that the correlations between CSF-S100B and GFAP levels were different among groups. The correlation of these proteins was stronger when the levels of CSF-GFAP were higher. The reason is probably because as an astrocytic protein, there is no actively secreted form of CSF-GFAP [17], but S100B is constantly released from astrocytes as a glial modulator implicated in the activity of a large number of targets [18, 20]. When astrocytes are severely damaged, large amounts of GFAP and S100B leak out. In this case, the leaking S100B becomes the main source of CSF-S100B. The CSF-S100B and GFAP levels reflect the degree of astrocytic injury and have a significant correlation with each other. However, when astrocytic injury is light, the leaking of S100B and GFAP decreases, and the proportion of leaking S100B in CSF-S100B decreases. As a result, the correlation between CSF-GFAP and S100B declines.

It was reported that the CSF-GFAP levels in NMOSD patients with only myelitis were higher than those in patients with only brain lesions [6]. As most of our NMOSD patients had combined lesions, very few patients were included in the analyses of influence by lesion site. However, we can still see a trend of higher levels of CSF-S100B in patients with myelitis than in patients with brain lesions, similar to CSF-GFAP. This is probably because a longer distance from the lesions to the puncture point and the majority of the CSF was reabsorbed before descending to the spinal subarachnoid space [51].

Some limitations in our study need to be addressed. First, our patient cohort was small, especially the MS group, and derived from a single university hospital. Second, very few patients in our study had monoregional lesions, and no patients had only optic lesions. Third, as not all patients had full data sets of neuroimaging, we did not analyze the lesion load, which may lead to bias in our results. As a result, further studies will be needed to validate our findings in larger cohorts. More patients with monoregional lesions, including ON, should be analyzed to investigate the influence of attack-related lesion sites on CSF-S100B and GFAP levels. The association between the lesion load and the CSF-S100B and GFAP levels should also be evaluated.

## 5. Conclusions

We present here for the first time a systematic comparative investigation of CSF-S100B and GFAP in NMOSD patients. Both the CSF-S100B and GFAP levels could discriminate NMOSD from MS and OND with high accuracy. However, the levels of CSF-S100B could distinguish AQP4-Ab–positive NMOSD from MOG-Ab–positive NMOSD with a higher accuracy than the GFAP levels and could also significantly discriminate MOG-Ab–positive patients from seronegative patients, while GFAP could not. Moreover, the CSF-S100B levels decreased more slowly and showed more clinical association than CSF-GFAP. Therefore, we suggest that although CSF-S100B lacks specificity compared with GFAP, it can still be used as a candidate tool for discriminating and evaluating NMOSD patients, especially for patients who present late after attack onsets.

## Figures and Tables

**Table 1:** Clinical characteristics of patients with NMOSD and MS.

Clinical characteristics	NMOSD			MS (n=12)
	AQP4 Ab+ (n=25)	MOG Ab+ (n=8)	Seronegative (n=16)	
Sex ratio, F (%)	24(96.0)	3(37.5)	11(68.8)	9(75.0)
Age, years, mean (SD)	44.7(17.0)	38.3(8.5)	42.1(11.4)	37.4(16.0)
Disease duration, months, mean (SD)	18.9(8.8)	13.3(8.7)	16.0(10.4)	22.4(7.3)
Site of the lesions, n (%)				
Optic nerve	15(60.0)	3(37.5)	7(43.6)	3(25.0)
Spinal cord	25(100.0)	6(75.0)	14(87.5)	7(58.3)
Cerebrum	11(44.0)	7(87.5)	8(50.0)	6(50.0)
Brainstem	13(52.0)	5(62.5)	10(62.5)	4(33.3)
EDSS, median (range)	6.5(2.0-8.5)	4.3(3.5-7.0)	6.3(2.5-8.0)	3.5(2.0-5.0)
CSF analysis at attack				
CSF WBC count, mean (SD), /ul	19.0(24.3)	32.0(47.8)	10.3(15.7)	9.7(10.7)
CSF protein level, mean (SD), mg/dl	47.8(26.8)	42.4(16.9)	39.0(20.0)	37.4(23.7)
CSF IgG index, mean (SD)	0.6(0.2)	1.1(1.6)	0.5(0.1)	0.6(0.3)
Positive oligoclonal bands, n (%)	15(60.0)	3(37.5)	9(56.3)	8(66.7)

Continuous variables are shown as the means (SD), and categorical variables are described as percentages. NMOSD, neuromyelitis optica spectrum disorder; AQP4 Ab+, aquaporin-4 antibody positive NMOSD; MOG-Ab+, myelin-oligodendrocyte glycoprotein antibody positive NMOSD; MS, multiple sclerosis; F, female; EDSS, Expanded Disability Status Scale; CSF, cerebrospinal fluid; WBC, white blood cell.

**Table 2:** Discriminating value of CSF-GFAP and CSF-S100B levels for NMOSD, MS, OND, and NMOSD subgroups.

**Diseases**	**Biomarker**	**AUC**	**Sensitivity (**%**)**	**Specificity (**%**)**	**Cut-off **	**95**%** CI**	***p*-Value**
NMOSD vs MS	S100B	0.839	71.4	91.7	241.4 pg/ml	0.74-0.94	<0.001
	GFAP	0.850	71.4	91.7	2.3 ng/ml	0.75-0.95	<0.001
NMOSD vs OND	S100B	0.823	65.3	100.0	300.2 pg/ml	0.72-0.92	<0.001
	GFAP	0.907	67.3	100.0	2.4 ng/ml	0.83-0.98	<0.001
MS vs OND	S100B	0.450	50.0	60.0	76.5 pg/ml	0.12-0.66	0.661
	GFAP	0.669	41.7	86.7	2.3 ng/ml	0.11-0.14	0.137
**Subgroups of NMOSD patients**							
AQP4 Ab+ vs MOG Ab+	S100B	0.865	84.0	100.0	282.5 pg/ml	0.74-0.99	0.002
	GFAP	0.772	72.0	100.0	6.2 ng/ml	0.61-0.93	0.022
AQP4 Ab+ vs Seronegative	S100B	0.587	36.0	100.0	2148.3 pg/ml	0.41-0.76	0.350
	GFAP	0.646	68.0	62.5	10.9 ng/ml	0.48-0.81	0.118
MOG Ab+ vs Seronegative	S100B	0.848	100.0	81.2	282.5 pg/ml	0.68-1.00	0.006
	GFAP	0.613	100.0	50.0	6.7 ng/ml	0.39-0.84	0.375

AUC, area under curve; CI, confidence interval; CSF, cerebrospinal fluid; GFAP, glial fibrillary acidic protein; NMOSD, neuromyelitis optica spectrum disorder; MS, multiple sclerosis; OND, noninflammatory neurological diseases; AQP4 Ab+, aquaporin-4 antibody positive NMOSD; MOG-Ab+, myelin-oligodendrocyte glycoprotein antibody positive NMOSD.

## Data Availability

The data used to support the findings of this study are available from the corresponding author upon request.

## Supplementary Materials

Supplementary Materials Supplementary Figure 1: ROC curves of CSF-S100B and GFAP levels for NMOSD subgroups, MS, and OND.
