# Supplementary material for: CSF-S100B Is a Potential Candidate Biomarker for Neuromyelitis Optica Spectrum Disorders
Source: Biomed Res Int. 2018 Oct 22;2018:5381239. doi: 10.1155/2018/5381239 (PMC6217894; doi:10.1155/2018/5381239)
Supplement: Supplementary Materials — Supplementary Figure 1: ROC curves of CSF-S100B and GFAP levels for NMOSD subgroups, MS, and OND. [file 5381239.f1.pdf]

Supplementary Figure 1. Receiver operator characteristics (ROC) curves of CSF-S100B and glial fibrillary acidic protein (GFAP) levels for discriminating neuromyelitis optica spectrum disorder (NMOSD) subgroups, multiple sclerosis (MS) and other noninflammatory neurological diseases (OND).

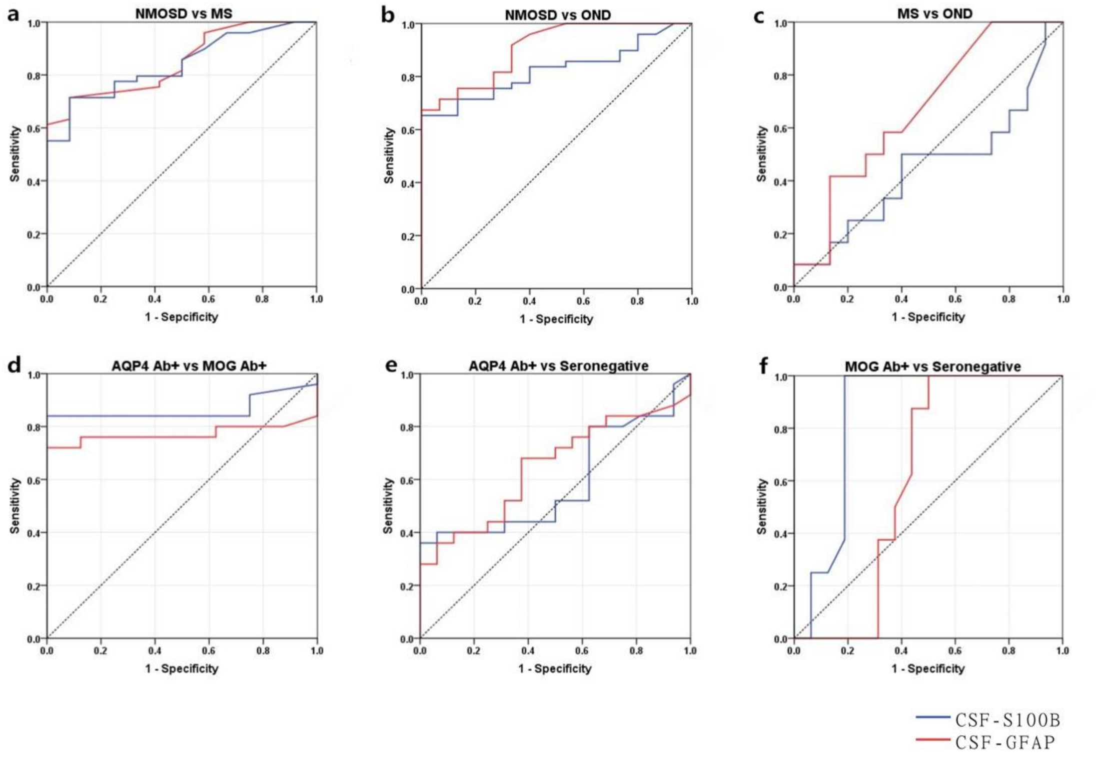

The S100B and GFAP levels significantly discriminated NMOSD from MS [95% confidence interval (CI) = 0.74-0.94 and 0.75-0.95, respectively] (a) and OND (95% CI = 0.72-0.92 and 0.83-0.98, respectively) (b) but failed to discriminate MS from OND (95% CI = 0.12-0.66 and 0.11-0.14, respectively) (c). Among subgroups of NMOSD, both the CSF-S100B and CSF-GFAP levels significantly differentiated patients with aquaporin-4 antibody-positive NMOSD (AQP4-Ab+) from patients with myelin-oligodendrocyte glycoprotein antibody-positive NMOSD (MOG-Ab+) (95% CI = 0.74-0.99 and 0.61-0.93, respectively) (d). Both the CSF-S100B and CSF-GFAP

levels could not differentiate AQP4-Ab+ NMOSD patients from seronegative NMOSD patients (95% CI = 0.41-0.76 and 0.48-0.81, respectively) (e). The CSF-S100B levels significantly discriminated MOG-Ab+ patients from seronegative patients (95% CI = 0.68-1.00), while CSF-GFAP could not (95% CI = 0.39-0.84) (f).
